# Selenium Accumulation, Speciation and Localization in Brazil Nuts (*Bertholletia excelsa* H.B.K.)

**DOI:** 10.3390/plants8080289

**Published:** 2019-08-16

**Authors:** Leonardo W. Lima, Gavin C. Stonehouse, Christina Walters, Ali F. El Mehdawi, Sirine C. Fakra, Elizabeth A. H. Pilon-Smits

**Affiliations:** 1 Department of Biology, Colorado State University, Fort Collins, CO 80523, USA; 2 National Laboratory for Genetic Resources Preservation, USDA-ARS, Fort Collins, CO 80521, USA; 3 Advanced Light Source, Lawrence Berkeley National Laboratory, Berkeley, CA 94720, USA

**Keywords:** Brazil nut, *Bertholletia excelsa* H.B.K., selenium, biofortification, toxicity, X-ray microprobe analysis, nutrition

## Abstract

More than a billion people worldwide may be selenium (Se) deficient, and supplementation with Se-rich Brazil nuts may be a good strategy to prevent deficiency. Since different forms of Se have different nutritional value, and Se is toxic at elevated levels, careful seed characterization is important. Variation in Se concentration and correlations of this element with other nutrients were found in two batches of commercially available nuts. Selenium tissue localization and speciation were further determined. Mean Se levels were between 28 and 49 mg kg^−1^, with up to 8-fold seed-to-seed variation (*n* = 13) within batches. Brazil nut Se was mainly in organic form. While present throughout the seed, Se was most concentrated in a ring 1 to 2 mm below the surface. While healthy, Brazil nuts should be consumed in moderation. Consumption of one seed (5 g) from a high-Se area meets its recommended daily allowance; the recommended serving size of 30 g may exceed the allowable daily intake (400 μg) or even its toxicity threshold (1200 μg). Based on these findings, the recommended serving size may be re-evaluated, consumers should be warned not to exceed the serving size and the seed may be sold as part of mixed nuts, to avoid excess Se intake.

## 1. Introduction

This study characterizes the chemical form of Selenium (Se) and its localization in the Brazil nut (*Bertholletia excelsa* H.B.K., Lecythidaceae), as well as the variation in Se concentration within and among different commercially available batches. *Bertholletia* is a monotypic tree genus in the Lecythidaceae family, and its only species, *B. excelsa*, produces large, oil-rich seeds. These, known as Brazil nuts, are of biological and nutritional interest, because they accumulate extraordinarily high Se levels. Selenium (Se) is an essential micronutrient for humans and other mammals. This element plays an important role in the organism, and its inadequate nutritional supplementation can cause a number of health disorders [1]. The main forms of Se found in humans are organic, in the form of the amino acids selenocysteine (SeCys), analog to cysteine (Cys), and selenomethionine (SeMet), analog to methionine (Met). SeCys is a structural part of the active site of twenty-five different selenoproteins [2], which play roles in the maintenance of physiological homeostasis, including the cellular redox state regulation and hormonal biosynthesis.

Adequate Se intake varies and depends on personal physiological and biological parameters such as body weight, age and sex [3,4]. Therefore, the Recommended Dietary Allowance (RDA) of Se in the United States and Canada ranges from 15 μg Se/day (infants from 0 to 6 months old) to 70 μg Se/day (women from 14 to 50 years old during lactation), while the recommendation for male and female adults, between 18 and 71 years old, corresponds to 55 μg Se/day [4]. The RDA varies in different countries [5]. The Austrian, German and Swiss nutrition societies recommend higher Se intake for adult women, 60 μg Se/day, and adult men, 70 μg Se/day [6], while in Japan the recommended Se intake is 25 μg Se/day for adult women and 35 μg Se/day for adult men [5].

Despite its importance to human metabolism, Se can become toxic above a certain threshold, due to its interference with sulfur (S) metabolism [7]. There is a narrow window between Se deficiency, adequacy and toxicity. The tolerable Se intake limit is considered 400 μg Se/day [4,8], while the intake of Se associated with toxicity (selenosis) was estimated to be around 1200 μg Se/day (in people exposed to large amounts of organic Se in China) [8]. Long-term exposure to moderate Se levels can results in chronic Se toxicity, and exposure to high Se levels can in some cases cause death due to acute toxicity. Chronic selenosis symptoms range from fragile or depigmented hair and nails to loss of these parts [9], and characteristic acute selenosis symptoms include diarrhea, nausea, skin rash, disorders to the nervous system, fatigue and irritability [4]. There are also possible risks of supra-nutritional Se levels, as suggested by several recent papers on the complex U-shaped relationship between Se dose and diseases such as type 2 diabetes or cancers [10,11,12].

While Se toxicity is a great concern, deficiency is an even bigger problem worldwide. Low dietary Se intake, less than 40 μg/day [13], is estimated to negatively affect more than one billion people worldwide [14] including areas in China, Eastern Europe, Brazil and Sub-Saharan Africa, Australia and New Zealand [3]. In low Se areas in China, two specific diseases related to Se deficiency occur, i.e., Keshan disease [13,15] and Kaschin-Beck disease [16,17]. The general symptoms of Se deficiency are related to impaired cellular redox capacity, thyroid function and immune defense [7,18,19]. Not surprisingly, several studies have found a positive correlation between Se deficiency and incidence of different types of cancer [20,21,22] as well as decreased survival in HIV-positive patients [7]. Deficiency symptoms may also include muscle weakness, muscle pain (myalgia) and heart dysfunction [23], irreversible brain injury and impaired fertility [7]. 

Selenium enters the food chain via plants, so an important source of Se to the human diet is plant-based food; this is particularly important for populations in low Se areas that rely on a vegetarian diet [5]. The Se concentration in crops varies greatly, not only due to species differences, but also due to variation in soil Se concentration worldwide, which is determined by geological processes [3]. In addition, local Se speciation and bioavailability are influenced by physico-chemical aspects of the soil [13]. The most common bioavailable Se forms are inorganic selenate (SeO_4_^2−^), found in well aerated, alkaline and oxidized soils, or selenite (SeO_3_^2−^), present in more acidic and reducing environments like wetlands [24]. Organic forms of Se, such as SeCys and selenomethionine (SeMet), analog to methionine (Met), can also be present in soil, through plant decomposition and microbiome activity; selenides and elemental Se can also be present, but are not very bioavailable [25,26].

To better provide Se to populations in low Se areas, different strategies are used to augment Se levels in crops, practices called biofortification [27,28,29,30]. An important factor in biofortification is the plant physiological capacity to take up, metabolize, translocate and accumulate Se. Plant species differ in their capacity to take up and assimilate Se. Selenium is not considered a nutrient for plants, but it is a beneficial element in low concentrations (~10 mg kg^−1^ dry weight, DW), owing to increased antioxidant capacity, which may lead to increased photosynthesis, stress resistance and ultimately growth [31]. Selenium can become toxic to plants at tissue levels above 100 mg kg^−1^ DW, because they non-specifically take up selenate (SeO_4_^2−^) via sulfate (SO_4_^2−^) transporters and assimilate it into the Se analogs of the amino acids Cys (SeCys) and Met (SeMet) [32]. The misincorporation of SeCys and SeMet results in protein malfunction and systemic oxidative stress [33]. 

While biofortification can overcome the physiological limitations of crop species, it usually requires costly fertilizer supplementation in agricultural areas with low soil Se concentration [3]. Naturally high-Se food sources can be a simple solution to the challenge of providing sufficient Se to populations in low-Se areas. Selenium accumulation capacity varies dramatically among plant species; in natural Se-containing areas the Se levels in vegetation can differ 100-fold [34]. Plants can be generally divided into three large groups based on Se content found in all their organs in natural environments: hyperaccumulators are plants that can exceed the threshold of 1000 mg Se kg^−1^ (DW), while secondary accumulators can accumulate from 100–1000 mg kg^−1^ DW and non-accumulators do not exceed 100 mg kg^−1^ DW [24]. Non-accumulators, i.e., most species, including crops, and secondary accumulators, e.g., *Brassica* crop species and several wild Brassicaceae and Asteraceae, tend to contain more inorganic Se, while hyperaccumulators such as *Stanleya pinnata* (Brassicacea) and *Astragalus bisulcatus* (Fabaceae) and *Lecythis ollaria* (Lecythidaceae, the monkey pot tree), typically sequester organic forms of Se that do not interfere with S metabolism and therefore are less toxic [35]. Selenium hyperaccumulation likely evolved independently in different plant families [36], and it is hypothesized to function in herbivory and pathogen protection as well as allelopathic interaction [37]. 

Among different plant-derived food Se sources, the Brazil nut (*B. excelsa* H.B.K), endemic to different countries in South America and a relative of the monkey pot tree, contains the highest reported concentration of Se among other nuts/seeds; a few Brazil nuts are sufficient to provide the Se RDA listed for North America, Asia and Europe [38]. Nuts are included in the healthy diet recommendations of several countries, due to their high nutritional value, fiber content, unsaturated fatty acids and minerals [39], however moderation consumption of nuts is advised due to high caloric values. Therefore, the regular intake of Brazil nuts could be recommended not only as a suitable strategy to prevent Se deficiency, but for several other health benefits such as anti-inflammatory properties, improvement of the cellular redox homeostasis and the reduced risk of different chronic diseases [39]. However, the Se content in these seeds can vary greatly according to soil properties with respect to Se concentration and bioavailability [38,40]. Since there is a very narrow window between adequate and toxic Se intake for humans, it is crucial to determine the variation in Se concentration within commercially available Brazil nut batches and also among batches from different companies, which could directly affect consumers. In addition, it is important to analyze the chemical forms of Se present in the seed, which could affect its nutritional value and potential toxicity to consumers. Last, it is also interesting to investigate any possible correlations between Se and other nutrients in the seed. 

In light of these considerations, this study characterized the chemical form of Se and its localization in the nut, as well as the variation in Se concentration within and among two different commercially available batches (hence referred to as A and B). Furthermore, levels of macronutrients and micronutrients in these nuts were characterized, and their interactions with Se investigated. These studies have significance for Brazil nut consumers, sellers and producers. This study also has intrinsic value; because this species has such unique properties with respect to Se, it is interesting to study its Se metabolic properties in detail.

## 2. Results and Discussion

### 2.1. Brazil Nut Selenium Concentration and Variation in Relation to Health 

There was significant variation in seed Se concentration within each one of the two Brazil nut batches from two different companies. A 2.5-fold difference between the lowest and the highest Se concentration for batch A and around a 8-fold difference for batch B, n = 13 per batch, (Table 1 and Table 2). The variation in Se concentration between these batches A and B was also more than 2-fold (Table 1 and Table 2). The Se levels ranged from 25 to 76 mg Se kg^−1^ in batch A and 10 to 79 mg Se kg^−1^ in batch B, while the averages were 49 and 28 mg Se kg^−1^, respectively (Table 1 and Table 2). These average levels are higher than the 19 ± 2.3 mg Se kg^−1^ reported to be present in Brazil nuts [41], and widely used as a public resource. They are also higher than the average level of 14.66 mg Se kg^−1^ found in another study using 72 nuts, however, the levels found here fell within the 0.2–253 mg Se kg^−1^ range that was reported [40]. To put these Se values into perspective, the RDA for the National Institutes of Health (NIH), U.S. Department of Health and Human Services, and also the U.S. Department of Agriculture (USDA) is 55 μg Se/day for adults. Consequently, the consumption of one seed (average of 5 g) from either batch A or batch B would most likely already meet or exceed this RDA. 

The commonly recommended serving size for Brazil nuts according to the NIH, the USDA and to the labeling on batch A and B bags, is ~30 g (corresponding to 6 seeds). The Se present in such a serving size would correspond to 1470 μg Se in batch A and 840 μg Se in batch B (considering the average Se per batch). The maximum allowable Se intake is considered 400 μg Se/day [4,8], while the intake of Se associated with toxicity is estimated to be around 1200 μg Se/day [8]. Thus, the amount of Se provided by the recommended 30 g serving size of these two analyzed batches of Brazil nuts, is 2- to 3.5-fold higher than the maximum allowable daily Se intake, and the Se in the serving size of batch A even exceeds the intake of Se associated with toxicity. In the more extreme scenario where a person would consume the entire 454 g bag of shelled Brazil nuts, the Se intake, calculated from the averages shown in the first paragraph, would amount to 22.2 mg Se and 12.7 mg Se for batches A and B, respectively, which is 10 to 20 times the toxic Se intake level.

Selenium toxicity is not only related to Sulfur metabolism dysfunction but can also trigger more intricate and wide responses in the organism. The consumption of high-Se Brazil nuts, containing 23 times higher than the RDA of 55 μg Se/day, was reported to be positively correlated with high expression of proinflammatory genes in obese woman, and the high concentration of Se in blood may increase the risk for different chronic diseases [42].

The two batches used in our study were from an unspecified region of Brazil. The biggest commercial Brazil nut producer in the world, made up of more than 1.2 million trees, is located in the region of Manaus, in the state of Amazonas. A large variation in Se concentration in Brazil nuts was reported [35], and this variation was correlated with the geographic origin in Brazil. The lowest mean concentrations (~2 mg Se kg^−1^) were found in the states of Acre and Mato Grosso, intermediate concentrations (~11 mg Se kg^−1^) in Roraima and the highest in the states of Amapá (51 mg Se kg^−1^) and Amazonas (68 mg Se kg^−1^). The Se variation in the seeds was correlated with variation in total soil Se concentration, which was also higher (~0.45 mg Se kg^−1^) in Amazonas than in the other states (~0.22 mg Se kg^−1^). In view of the finding that the world’s main Brazil nut producer is in a Se-rich area, and that the Se levels were high in our two tested Brazil nut batches, it is reasonable to assume that most commercially available Brazil nuts originating from Brazil could potentially be high in Se. These findings are important to consumers and sellers, because the commercialized products usually do not specify the Brazilian region of origin. In addition, there is substantial variation from seed to seed, possibly caused by genetic variation between trees or by local variation in soil Se concentration or in Se bioavailability due to soil acidity [35].

### 2.2. Selenium Localization and Speciation in Brazil Nuts Using X-ray Microprobe Analysis 

**Figure 1:**
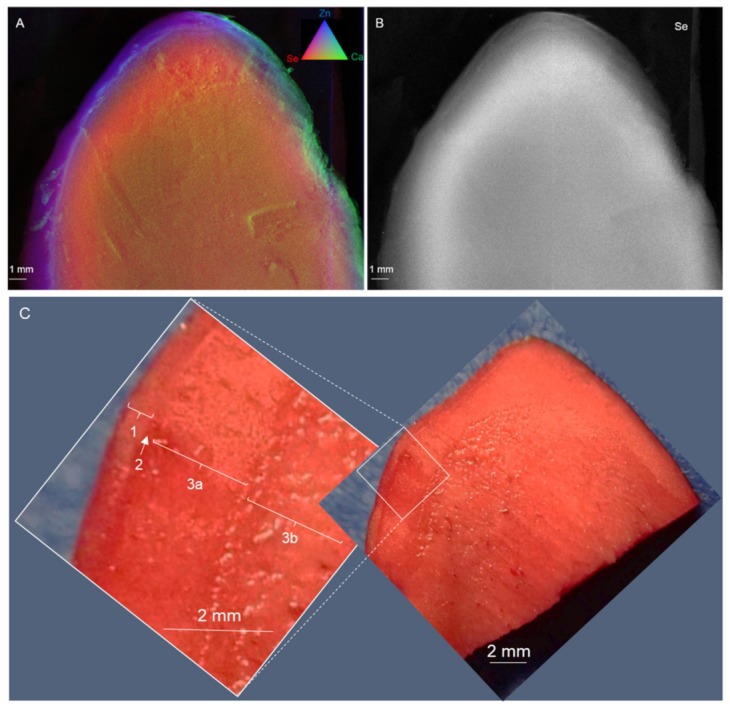
Micro X-ray fluorescence elemental distribution maps of a longitudinal section of Brazil nut #13 (25 mg Se kg^−1^, Table 1). Se is shown in red (**A**) or white (**B**). Panel **A** also shows Zn in blue and Ca in green. Panel (**C**) shows a longitudinal section of another Brazil nut stained with triphenyl tetrazolium (red); Numbered tissue layers are discussed in the text.

In addition to the concentration of total Se, it is important to consider the chemical forms of Se in the Brazil nuts, because these differ in nutritional value. Supplementation with organic forms of Se has been reported to be more effective compared to inorganic forms [7,43]. Micro X-ray fluorescence (XRF) was used to investigate Se localization in the Brazil nuts. First, a longitudinally cut seed was analyzed (Figure 1A,B). The Se was present throughout the seed, with strongest concentration in a tissue layer along the periphery, 1 to 2 mm below the surface. Outside of this high-Se zone, a high Zn concentration was present along the outer 1 mm of the seed, while Ca was most concentrated at the extreme exterior (Figure 1A). 

The vital staining with triphenyl tetrazolium chloride (Figure 1C) shows that all of the analyzed tissues in the embryo were alive, and also revealed the unusual seed anatomy of this species, which has been described earlier [44]. Most of the tissues were reported to consist of undifferentiated embryo cells, surrounded by a thin layer of tubular cells, possibly endosperm [45], and covered by a hard, lignified testa (mostly non-living cells). The embryo is classified as macropodial, in which the cotyledons are very rudimentary, even if present, and most of the tissue is considered to be the hypocotyl [46,47]. The outermost cells of the hypocotyl have large oil bodies and surround a procambium ring which is four to six cell layers thick. The ring of cells forming the procambium is the only evidence of a meristematic region within the embryo. Cells of the inner core of the embryo, comprising most of the volume, are undifferentiated parenchyma [48]. 

In Figure 1C, the endosperm may correspond with tissue #1; the thin layer of cells below it (#2, indicated by an arrow) may correspond to the procambium ring (meristematic tissue giving rise to vascular tissues in the seedling), and tissues #3a and b may be the undifferentiated parenchyma of the hypocotyl, making up most of the embryo. The area of concentrated Se appears to be along the outside of the embryo tissues, just below the endosperm, in tissue #3A (undifferentiated parenchyma) and possibly tissue #2 (procambium) (Figure 1B,C). Therefore, we speculated that the Se accumulates in such a way that it can readily be distributed to the growing meristems during seed germination. This may serve to protect these tissues from biotic stresses. Selenium has been found to protect the plant from herbivores and pathogens, also at the levels found in these nuts [37]. 

**Figure 2:**
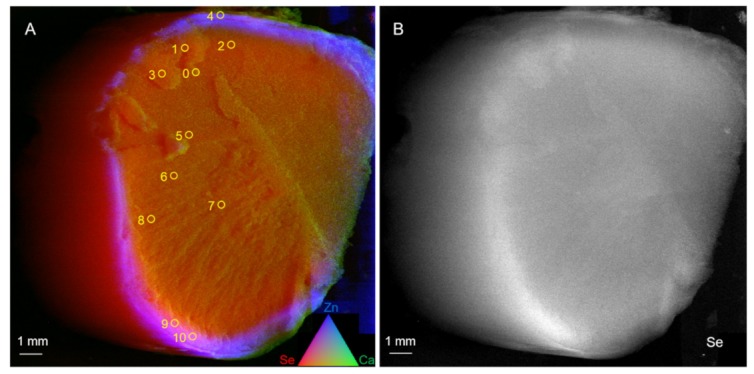
Micro X-ray fluorescence elemental distribution maps of a cross section of Brazil nut #12 (48 mg Se kg^−1^, Table 1). Se is shown in red (**A**) or white (**B**). Panel **A** also shows Zn in blue and Ca in green. Micro X-ray absorption near-edge structure spot locations are shown as numbered yellow circles; speciation results are shown in Table 3.

Further μXRF analysis of a different, cross-cut Brazil nut showed a ring-shaped Se concentration 1 to 2 mm from the seed’s exterior, in agreement with the distribution found in the longitudinally cut seed (Figure 2).

**Figure 3:**
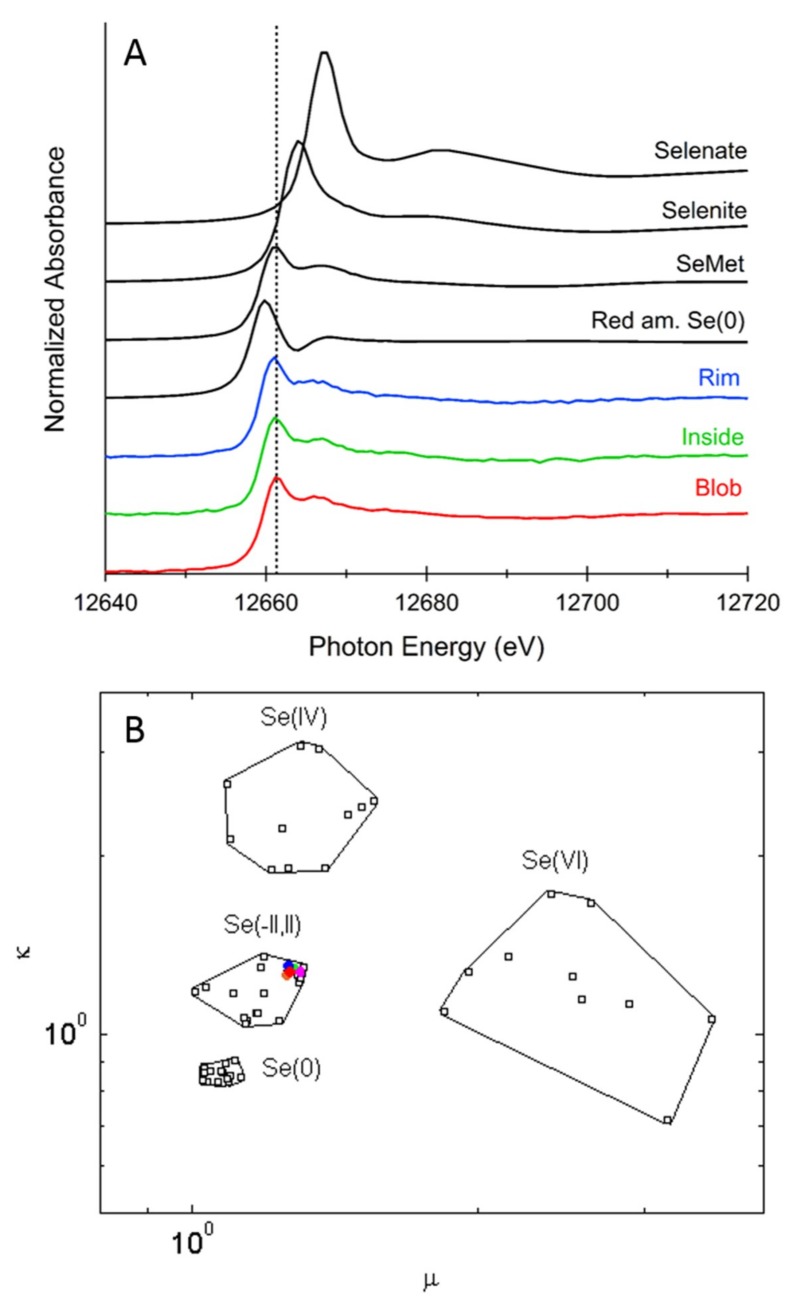
(**A**) Se K-edge micro X-ray absorption near-edge structure spectra of Brazil nut at locations shown in Figure 2A. The “Blob” (red graph) is the average spectrum of spots 0, 1 and 3, the “Inside” (green graph) is the average spectrum of spots 6 and 7 and the “Rim” (blue graph) is the average spectrum of spots 9 and 10. Spectra of selected standard compounds are shown in black for comparison. (**B**) Se valence scatter plot of the Brazil nut X-ray absorption near-edge structure data (same color as in panel A), plus spot 4 is in magenta and spot 2 is in orange. Se standard compounds are shown as open black squares.

At the locations indicated (Figure 2), 11 micro X-ray absorption near-edge structure (μXANES) spectra were collected across this seed, for Se speciation analysis (Figure 3A). Se valence scatter plots of the μXANES data along with 52 standard compounds showed that Se throughout the Brazil nut was mainly in organic forms (Figure 3B). Least squares combination fitting further revealed that the Se in the seed consisted predominantly (91% on average) of organic C-Se-C species, in all tested areas (Table 3); other, minor forms of Se were fitted as elemental Se (Se^0^) and Se (IV) species. The detected C-Se-C compounds may include selenomethionine (SeMet), methyl-selenocysteine (MetSeCys) and/or Se-lanthionine, which are indistinguishable by μXANES. The SeMet form, could either be present as a free amino acid or incorporated into proteins, which is also indistinguishable by μXANES. Studies [49,50,51], that used combinations of liquid chromatography and mass spectrometry, found the main form of Se in Brazil nuts to be SeMet. These studies detected this form after proteinase K treatment, suggesting that SeMet was incorporated into proteins. These findings were in agreement with our μXANES data. Incorporation of SeMet in proteins is non-specific, replacing Met, and this is less toxic to organisms than non-specific SeCys incorporation in proteins, or the accumulation of inorganic forms of Se [7,24]. For mammals, SeMet is a good source of dietary Se, whether incorporated into protein, or as a free amino acid [7,19,43].

### 2.3. Interactions of Se With Other Elements in Brazil Nut 

A few significant correlations were found between Se and other elements (Table 4, Table 5 and Table 6, *p* < 0.05 levels in bold). In batch A (Table 4), Se was negatively correlated with magnesium (Mg, R = 0.47); some patterns (non-significant, NS) were also found supporting a negative relationship of Se with phosphorus (P), calcium (Ca), zinc (Zn) and iron (Fe). In contrast, batch B (Table 5) showed no significant correlations with Se and other elements, only a NS tendency for Se to positively interact with Zn, Fe and nickel (Ni). Across both batches (Table 6), Se was positively correlated with sulfur (S, R = 0.53) and copper (Cu, R = 0.42), and there was a NS pattern of positive interaction with Ni. These elements were all present at higher levels in batch A than B, by 34% (S), 85% (Cu) and 42% (Ni), respectively (Table 1 and Table 2). Among the other nutrients in the seeds, there were consistent positive correlations (*p* < 0.05) between the levels of Mg, Zn, Fe, and P (Table 4, Table 5 and Table 6). 

Thus, apart from the negative correlation between Se and Mg in batch A, there was no evidence that Se accumulation in the Brazil nuts may negatively affect levels of other healthy nutrients for consumers. Although across both batches, there was a positive correlation between Se, S and Cu, there were no such correlations within batches A or B. Rather, this correlation could be explained by differences between the batches: batch A had higher levels than batch B for Se, S, and Cu. Molybdenum (Mo) levels were also higher in batch A, but no statistics could be done because the levels were too low to be detectable in batch B. In this context it was interesting to note that Se, S and Mo were similar oxyanions that could be taken up by the same transporters [35].

## 3. Materials and Methods 

### 3.1. Biological Material 

Two different samples of commercially available in-shell Brazil nuts (*B. excelsa* H.B.K.) were used in this experiment. Both 454 g bags listed Brazil as the country of origin. The first batch was purchased from a U.S.A. website (company A) specializing in nuts, hence referred to as “batch A”. The second batch was purchased in a local store from a big U.S.A. supermarket franchise (company B), hence referred to as “batch B”. Thirteen seeds from each batch were randomly picked for elemental composition analysis. All were in good condition for consumption (no indication of browning or other degradation). 

### 3.2. Elemental Composition

Fresh samples of 13 different Brazil nuts samples from each seed batch were weighed to 100 mg and dried at 50 °C until constant weight. These samples were then digested with 1 mL of nitric acid [52]; samples were heated for 2 h at 60 °C and 6 h at 125 °C, then diluted to 10 mL with distilled water. Inductively coupled plasma optical emission spectroscopy (ICP-OES) was used to analyze the digested seed samples’ elemental composition (K, P, S, Mg, Ca, Cu, Zn, Fe, Mn, Ni and Mo).

### 3.3. Selenium Localization and Speciation

Selenium (and calcium, zinc) localization and speciation were analyzed in two different biological replicates of Brazil nut samples, from batch A (seed #12 and #13), using X-ray microprobe imaging [53]; batch B was not yet available at the time. Analyses were performed at beamline 10.3.2 (X-ray Fluorescence Microprobe) of the Advanced Light Source (ALS), at Lawrence Berkeley National Lab (Berkeley, CA, USA) using a Peltier cooling stage (−25 °C). Localization of Se, Ca and Zn was determined on a longitudinal section of sample #13 and a cross section of sample #12, cut fresh with a single-edged carbon steel blade, and then kept frozen during analysis. Micro-focused X-ray fluorescence (μXRF) maps were recorded at 13 keV incident energy, using 20 µm × 20 µm pixel size, a beam spot size of 7 µm × 7 µm, using 70 ms dwell time (Figure 1) and 50 ms dwell time (Figure 2). Maps were then deadtime-corrected and decontaminated. Selenium K-edge micro X-ray absorption near-edge structure (μXANES) spectroscopy (in the range 12,500–13,070 eV) was used to analyze Se speciation on eleven different spots on sample #12, close to areas showing high Se concentration in the μXRF maps. Because of time constraints, only one of the nuts could be analyzed for speciation, and the cross-section was chosen because it would give information about speciation in different tissues. Spectra were energy calibrated using a red amorphous Se standard, with the main peak set at 12,660 eV. Least-square linear combination fitting of the μXANES data was performed in the range of 12,630 to 12,850 eV using a library of 52 standard selenocompounds and procedures described by Fakra [54]. Additionally, a selenium valence scatter plot where kappa and mu represent the normalized absorption values at 12,664.25 and 12,667.8 eV respectively, was extracted from the Brazil nut μXANES data, using procedures described by Németh [51]. All data were recorded in fluorescence mode using a 7-elements Ge solid state detector (Canberra, ON) and processed using custom LabVIEW programs available at the beamline.

### 3.4. Triphenyl Tetrazolium Staining

Triphenyl tetrazolium staining on randomly selected Brazil nuts was performed according to Miller [55]. Representative results from one longitudinally cut seed from batch A is shown.

### 3.5. Statistical Analysis 

The software JMP-IN 13.0.0 (SAS Institute, Cary, NC, USA) was used for statistical data analysis. Student t-test was used to compare batch A with B. Bivariate analyses (Fit x by y) were performed to determine correlations between elements, and the correlation coefficients (R) are shown in Table 4, Table 5 and Table 6. Linear fit was then used to analyze variance and to determine the p-values, which were then plotted in Table 4, Table 5 and Table 6.

## 4. Conclusions

This study analyzed the variation in Se concentration, as well as Se tissue localization and chemical speciation, and the relation of Se with other nutrients in 26 seeds in two different commercially available Brazil nut batches, 13 seeds per batch. Several important findings that are of basic biological interest are presented. The Se was found to be present in a tissue layer 1 to 2 mm below the seed surface, along its periphery. Based on µXANES fitting, the forms accumulated were organic C-Se-C compound(s) that may include SeMet, MetSeCys and/or Se-lanthionine. Together, this information provides novel insight into Se physiology and metabolism in this extraordinary Se-accumulating plant species. The findings also have significance for Brazil nut consumers, producers, sellers, and regulatory agencies. Brazil nuts contain the highest Se levels of any plant-based food source [35,36,42], and are therefore a valuable source of this essential micronutrient. However, Se can easily become toxic at elevated levels, and thus it is vital to inform and protect consumers from possible toxic effects of overconsumption of these high-Se seeds. 

The anatomy of *B. excelsa* seeds is unusual: it consists almost entirely of embryo hypocotyl parenchyma, with a thin layer of endosperm around it and a meristematic layer in between (procambium). The area of concentrated Se appears to be below the endosperm along the outside of the embryo hypocotyl, corresponding with the outer parenchyma layer, and possibly the procambium. The Se in the Brazil nuts was mainly organic, reported to be the most effective dietary source of Se [7]. There was large seed-to-seed variation (up to 8-fold) in Se concentration and the averages between the batches was ~2-fold. The levels of Se found were such that the consumption of one seed (5 g) was enough to meet or even exceed the recommended daily allowance (RDA) for Se.

While healthy, Brazil nuts should be consumed in moderation, it is important to emphasize that the levels of Se found in these two batches were high enough to exceed the maximum allowable daily intake of Se (400 μg) if consumed at the recommended serving size of 30 g (6 seeds). Depending on the batch, the recommended serving size may even exceed the Se intake level reported to cause toxicity symptoms (1200 μg). Therefore, unless low Se levels in batches of seeds can be demonstrated, it would be safer for the recommended serving size for Brazil nuts to be reduced to 15 g (3 seeds) to ensure safe Se intake, and to warn consumers to not exceed this limit. In addition, it would be helpful to include on the package the geographic origin of the Brazil nut and ideally the Se concentration of the specific batch, with an indication of the % of RDA for Se. Furthermore, to avoid Se toxicity due to overconsumption, the package size of Brazil nuts from high-Se geographic areas may be reduced, or these seeds could preferentially be sold as part of mixed nuts packages. 

## Figures and Tables

**Table 1:** Elemental composition of 13 commercially available Brazil nuts (Batch A), imported from Brazil.

Seed#	Selenium (mg/kg)	Macronutrients (mg/g)					Micronutrients (mg/kg)					
		K	P	S	Mg	Ca	Cu	Zn	Fe	Mn	Ni	Mo
**1**	**48.6**	5.6	4.6	3.9	2.2	1.9	34.8	23.8	3.7	6.9	7.0	0.8
**2**	**56.7**	6.4	6.4	3.8	2.3	0.8	56.0	18.7	2.5	5.1	3.5	1.1
**3**	**30.0**	9.3	5.5	3.0	2.3	1.1	28.2	11.7	1.2	4.4	4.4	2.4
**4**	**47.0**	6.7	6.6	4.7	3.0	0.3	18.1	29.5	6.9	3.7	6.2	1.0
**5**	**40.3**	7.5	7.9	4.5	2.7	1.0	46.2	16.1	3.5	5.5	2.8	ND
**6**	**64.9**	8.3	6.4	4.8	2.6	0.7	28.7	32.4	2.0	7.0	1.9	1.8
**7**	**74.0**	3.6	5.5	3.3	2.0	1.0	22.1	15.2	1.9	7.0	2.4	1.0
**8**	**58.4**	7.2	5.8	4.0	2.2	0.5	33.8	13.6	2.8	3.3	4.6	2.8
**9**	**75.5**	7.8	7.7	3.7	2.0	1.4	19.5	12.9	4.1	12.2	2.9	1.5
**10**	**47.3**	5.7	6.0	4.2	2.3	1.3	22.8	12.8	4.3	4.6	1.3	3.7
**11**	**24.4**	5.7	8.2	2.6	2.9	1.6	19.4	33.9	12.5	8.5	2.2	ND
**12**	**48.0**	7.8	6.6	3.4	2.8	1.0	27.6	36.9	19.6	5.8	2.7	0.3
**13**	**24.9**	7.9	10.3	4.1	3.2	2.5	16.9	47	25.5	7.2	2.3	ND
**Mean**	49 *	**6.9**	**6.7**	3.9 *	**2.5**	**1.2**	28.8 *	**23.4**	**7.0**	**6.2**	**3.4**	**1.2**
**SD**	**16**	**1.5**	**1.5**	**0.6**	**0.4**	**0.6**	**11.4**	**11.2**	**7.4**	**2.3**	**1.7**	**1.2**
**Range**	**25–76**	**3.6–9.3**	**4.6–10.3**	**2.6–4.8**	**2.0–3.2**	**0.5–2.6**	**17–56**	**12–47**	**1.2–26**	**3.3–12.2**	**1.3–6.2**	**0.3–3.7**

Seeds 12 and 13 were used for XRF and XANES analysis. ND = not detectable. Asterisks denote significant differences between batch A and B, (*t*-test, *p* < 0.05).

**Table 2:** Elemental composition of 13 commercially available Brazil nuts (Batch B), imported from Brazil.

Seed#	Selenium (mg/kg)	Macronutrients (mg/g)					Micronutrients (mg/kg)					
		K	P	S	Mg	Ca	Cu	Zn	Fe	Mn	Ni	Mo
**14**	**12.1**	5.8	6.2	2.9	2.6	0.6	13.2	26.3	7.0	5.4	2.2	ND
**15**	**17.4**	7.7	6.1	2.7	2.3	0.4	12.6	22.4	10.7	4.5	2.9	ND
**16**	**18.1**	5.7	7.9	2.8	2.9	0.9	21.7	28.1	9.7	10.0	1.2	ND
**17**	**11.9**	4.8	6.1	3.2	2.6	1.3	14.2	37.1	6.9	2.7	1.5	ND
**18**	**78.7**	4.9	7.8	3.2	3.1	1.2	16.5	46.6	12.0	5.1	4.6	ND
**19**	**16.4**	4.3	5.7	2.1	2.2	1.9	19.2	22.4	5.0	8.9	2.9	ND
**20**	**15.1**	6.0	7.8	4.3	3.7	1.0	15.8	47.3	12.5	3.7	2.3	ND
**21**	**12.6**	6.3	3.6	1.6	1.3	0.6	11.2	9.6	2.3	2.6	0.9	ND
**22**	**17.0**	10.8	7.4	2.7	2.8	1.9	20.8	28.5	5.6	4.7	3.9	ND
**23**	**23.1**	7.0	5.1	2.4	2.4	0.7	16.2	21.3	2.3	5.7	2.2	ND
**24**	**10.0**	6.2	7.3	2.6	2.9	1.3	12.2	38.8	8.8	12.1	2.3	ND
**25**	**43.7**	5.8	7.9	3.4	3.4	1.0	12.3	38.2	13.7	5.7	1.0	ND
**26**	**27.7**	9.7	6.0	2.2	2.8	0.8	15.0	15.1	1.9	2.9	2.7	ND
**Mean**	27.7 *	**6.6**	**6.5**	2.8 *	**2.7**	**1.1**	15.6 *	**29.6**	**7.6**	**5.7**	**2.4**	**—**
**SD**	**19.0**	**1.9**	**1.3**	**0.7**	**0.6**	**0.5**	**3.4**	**11.8**	**4.1**	**3.0**	**1.1**	**—**
**Range**	**10.0–78.7**	**4.3–10.8**	**3.6–7.9**	**1.6–4.3**	**1.3–3.7**	**0.4–1.9**	**11.2–21.7**	**9.6–47.3**	**1.9–13.7**	**2.6–12.1**	**0.9–4.6**	**—**

ND = not detectable. Asterisks denote significant differences between batch A and B (*t*-test, *p* < 0.05).

**Table 3:** Selenium speciation in seed #12 as determined by least-square linear combination fitting of the Micro X-ray absorption near-edge structure (µXANES) spectra collected at locations shown in Figure 2A. NSS = normalized sum of squares. C-Se-C may correspond to the organic forms SeMet, MeSeCys and/or Se-lanthionine, which are indistinguishable by µXANES. Errors on fits are +/−10%. N.D: Not detected. Note: The spot 4 spectrum was too noisy to fit, so is not shown in the table.

XANES Spots	NSS (×10^−4^)	C-Se-C	Se (IV)	Se (0)
Avg 0,1,3 (“Blob”)	3.4	100%	N.D.	N.D.
2	5.2	100%	N.D.	N.D.
5	5.8	64%	10%	26%
Avg 6,7 (Inside)	5.8	100%	N.D.	N.D.
8	6.7	100%	N.D.	N.D.
Avg 9,10 (Rim)	4.1	81%	5%	14%

**Table 4:** *p*-values for positive (+) and negative (−) correlations between nutrient concentrations in Brazil nut batch A (*n* = 13).

Nutrients	Se	K	P	S	Mg	Ca	Cu	Zn	Fe	Mn	Ni
**K**	0.368										
**P**	(−) 0.146	0.351									
**S**	0.439	0.550	0.879								
**Mg**	**(−) 0.010^1^**	0.341	**(+) 0.012^2^**	0.527							
**Ca**	(−) 0.133	0.891	(+) 0.095	0.349	0.447						
**Cu**	0.735	0.871	0.367	0.599	0.406	0.334					
**Zn**	(−) 0.134	0.617	**(+) 0.039^3^**	0.804	**(+) 0.004^5^**	(+) 0.178	0.257				
**Fe**	(−) 0.065	0.580	**(+) 0.007^4^**	0.645	**(+) 0.004^6^**	**(+) 0.047^7^**	(−) 0.170	**(+) 0.0003^8^**			
**Mn**	0.364	0.891	(+) 0.197	0.370	0.626	(+) 0.083	0.266	0.673	0.616		
**Ni**	0.924	0.992	(−) 0.113	0.713	0.739	0.591	0.594	0.708	0.427	0.309	

The +/− values are shown for *p* < 0.20; correlations significant at the 0.05 level are in bold. Exponential numbers refers to correlation coefficient (R): **1** = 0.68; **2** = 0.67; **3** = 0.57; **4** = 0.70; **5** = 0.83; **6** = 0.74; **7** = 0.55; **8** = 0.84.

**Table 5:** *p*-values for positive (+) and negative (−) correlations between nutrient concentrations in Brazil nut batch B (*n* = 13).

Nutrients	Se	K	P	S	Mg	Ca	Cu	Zn	Fe	Mn
**K**	0.612									
**P**	0.204	0.933								
**S**	0.445	0.467	**(+) 0.004** ^**1**^							
**Mg**	0.236	0.980	**(+) 0.0001** ^**2**^	**(+) 0.0002^5^**						
**Ca**	0.909	0.970	0.285	0.882	0.527					
**Cu**	0.859	0.635	0.223	0.889	0.496	**(+) 0.048** ^**10**^				
**Zn**	(+) 0.171	0.209	**(+) 0.001** ^**3**^	**(+) 0.0001** ^**6**^	**(+) 0.008** ^**8**^	0.301	0.8431			
**Fe**	(+) 0.183	0.224	**(+) 0.002** ^**4**^	**(+) 0.001** ^**7**^	**(+) 0.008** ^**9**^	0.992	0.8494	**(+) 0.001^11^**		
**Mn**	0.714	0.337	0.228	0.745	0.632	0.304	0.3481	0.550	0.506	
**Ni**	(+) 0.105	0.325	0.364	0.806	0.517	(+) 0.172	0.2344	0.425	0.840	0.942

The +/− values are shown for *p* < 0.20; correlations significant at the 0.05 level are in bold. Exponential numbers refers to correlation coefficient (R): **1** = 0.74; **2** = 0.90; **3** = 0.80; **4** = 0.78; **5** = 0.85; **6** = 0.86; **7** = 0.79; **8** = 0.81; **9** = 0.69; **11** = 0.79.

**Table 6:** *p*-values for positive (+) and negative (−) correlations between nutrient concentrations in Brazil nut batches A+B (*n* = 26).

Nutrients	Se	K	P	S	Mg	Ca	Cu	Zn	Fe	Mn
**K**	0.636									
**P**	0.909	0.544								
**S**	**(+) 0.005** ^**1**^	0.875	(+) 0.094							
**Mg**	0.490	0.734	**(+) 0.0001** ^**3**^	(+) 0.116						
**Ca**	0.624	0.945	**(+) 0.0327** ^**4**^	0.886	0.438					
**Cu**	**(+) 0.029** ^**2**^	0.579	0.794	**(+) 0.0143** ^**7**^	0.359	0.906				
**Zn**	0.471	0.424	**(+) 0.0005** ^**5**^	0.356	**(+) 0.0001** ^**8**^	(+) 0.119	(−) 0.104			
**Fe**	0.418	0.822	**(+) 0.0001** ^**6**^	0.612	**(+) 0.0006** ^**9**^	(+) 0.057	(−) 0.130	**(+) 0.0001** ^**11**^		
**Mn**	0.615	0.378	(+) 0.063	0.759	0.915	**(+) 0.0419** ^**10**^	0.850	0.545	0.457	
**Ni**	(+) 0.085	0.448	0.453	(+) 0.140	0.879	0.756	(+) 0.072	0.735	0.403	0.559

The +/− values are shown for *p* < 0.20; correlations significant at the 0.05 level are in bold. Exponential numbers refers to correlation coefficient (R): **1** = 0.53; **2** = 0.42; **3** = 0.74; **4** = 0.42; **5** = 0.63; **6** = 0.70; **7** = 0.47; **8** = 0.81; **9** = 0.63; **10** = 0.40; **11** = 0.77.
